# An Investigation of the Effectiveness of Arts Therapies Interventions on Measures of Quality of Life and Wellbeing: A Pilot Randomized Controlled Study in Primary Schools

**DOI:** 10.3389/fpsyg.2020.586134

**Published:** 2020-12-15

**Authors:** Zoe Moula, Joanne Powell, Vicky Karkou

**Affiliations:** Research Centre for Arts and Wellbeing, Edge Hill University, Lancashire, United Kingdom

**Keywords:** arts therapies, art therapy, music therapy, dance movement therapy, dramatherapy, randomized controlled study, child, primary school arts therapies in primary schools

## Abstract

**Background:**

Over the last decades there has been a change in the way schooling is perceived recognizing that children’s learning is closely linked to children’s health. Children spend most of their time at school, which is often the place where problems are identified and interventions are offered, not only for treatment but also prevention. Embedding arts therapies into the educational system may help address children’s emerging needs and have a positive impact on their wellbeing.

**Methods:**

A pilot cross-over randomized controlled design was employed to investigate the effectiveness of an arts therapies intervention on a series of child- and teacher-reported outcome measures, specifically, health related quality of life (assessed using a HRQOL scale; EQ-5D-Y), wellbeing and life functioning (assessed using the child outcome rating scale; CORS), emotional and behavioral difficulties (assessed using the strengths and difficulties questionnaire; SDQ), as well as duration of sleep (assessed using Fitbits). Sample size calculations for future large-scale studies were also performed, and the sustained impact of the intervention was evaluated at 3, 6, and 12 months follow-up. The pluralistic theoretical and therapeutic framework of this intervention was informed by a systematic review on school-based arts therapies interventions and is presented in detail in the study protocol. Participants were 62 children with mild emotional and behavioral difficulties.

**Results:**

Improvements in HRQOL and CORS were greater in those engaged in the arts therapies intervention than the control groups and were maintained at the follow-up stages. Significant improvements were only found for duration of sleep (*P* = 0.002) and SDQ (*P* = 0.008). Minimal clinically important differences (MCIDs) as defined in the published protocol were found for CORS, SDQ and duration of sleep, but not HRQOL.

**Discussion:**

Findings indicate that the arts therapies interventions were having a clinically significant effect on life functioning, duration of sleep, emotional and behavioral difficulties. Findings also indicate a small effect size for health related quality of life, suggesting the intervention was having a small positive effect on this outcome measure. The study indicates that all outcome measures assessed here would be suitable for inclusion in a larger randomized controlled study utilizing these arts therapies interventions, and that a sample size of 225 participants would be required if these outcome measures were used.

## Introduction

It is increasingly recognized that children’s learning is closely linked to their health. As a result, within UK schools, there are several multi-agency collaborations to work with complex factors affecting children’s lives. As children spend a great deal of their day at school, it is often the place where problems can be identified and interventions can be offered in an appropriate and timely fashion. A prevention or resilience model suggests that early interventions are important as a way of supporting children cope with daily challenges before more serious mental health problems arise. Such approaches may also prepare children to deal with problems they may face in the future. For this reason, embedding arts therapies into the educational system may help to address children’s emerging needs and have a positive impact on their wellbeing.

The term ‘arts therapies’ refers to art, music, drama and dance movement therapy, psychotherapeutic approaches that aim to facilitate psychological change and personal growth through the use of arts media. Arts therapies have been defined as, “*the creative use of artistic media for non-verbal and/or symbolic communication, within a holding environment, encouraged by a well-defined client-therapist relationship, in order to achieve personal and social therapeutic goals appropriate for the individual*” (Karkou and Sanderson, 2006, p. 47). In this intervention, groups received a single arts therapy discipline (i.e., art therapy or music therapy), known as creative arts therapies or in the United Kingdom as arts therapies/arts psychotherapies, rather than a combination of the different disciplines, known as expressive arts therapies (Karkou and Sanderson, 2006). In the United Kingdom, art therapy, music therapy, and dramatherapy are recognized professional bodies regulated by the Health and Care Professions Council (HCPC), while dance movement psychotherapy is regulated by the United Kingdom Council for Psychotherapy (UKCP). All four arts therapies work with a range of client groups in a variety of settings, such as hospitals, clinics, outpatient treatment facilities, shelters, and schools (Karkou, 2010).

Arts therapies have been used widely for more than half a decade for children and young people with a wide range of needs: from mild emotional or behavioral difficulties to experiences of trauma, abuse, bereavement, migration or diagnosed difficulties, such as ADHD and ASD. More recently, arts therapists have seen a substantial growth of their employment in educational settings to alleviate and prevent transition related difficulties. For example, in order to prepare children for hospitalization, transition from primary to secondary schools, from special to mainstream education, or transition resulting from a change in family circumstance, such as the death of a parent, parental separation or divorce (Pellegrini, 2011). It is therefore possible that trained and qualified arts therapists can facilitate children’s personal change and growth, bridging the gap between health and education.

It is estimated that more than half of all registered arts therapists in the United Kingdom are working with children and young people (Karkou, 2010), which may vary for the different types of arts therapies. In music therapy, the latest workforce survey (Carr et al., 2017) showed that schools were the most commonly reported setting, while working with children and adolescents was the most commonly reported post (78% of all posts). In art therapy, a current workforce survey (British Association of Art Therapists (BAAT), 2018) showed that 68% of all art therapies were working with children and young people, while 35% of them were based in school-based settings. In dance movement therapy, it was estimated that 33% of dance movement therapists were working with children and young people, while schools were the third most commonly reported setting (28% of all posts) (Zubala and Karkou, 2014). Following contact with the British Association of Dramatherapists in 2020, it was estimated that approximately half of dramatherapists in the United Kingdom were working with children and young people, the majority of whom were based in school-based settings. Nevertheless, the inclusion and integration of arts therapies into regular mental health provision in educational settings has only recently began. Underpinning arts therapies practice with solid research will support and strengthen such an integration (Uttley et al., 2015).

One of the challenges that school mental health services deal with is how to evaluate and monitor changes in children’s life (Camhs review, 2012). It is being increasingly recognized that children have substantial insight into their own wellbeing, and that measuring outcomes of health improvement need to examine children’s perceptions (Sofer and Ben-Arieh, 2014; Ben-Arieh and Kosher, 2018; Andresen et al., 2019). The first international study on children’s subjective well-being, the ‘Children’s Worlds,’ collected data from more than 17,000 children and found that children as young as eight are aware of their own needs (Rees et al., 2016). Therefore, there is a strong argument that efforts to improve children’s wellbeing need to include their own voice.

This has led to an increased interest and worldwide recognition of Participant-Reported Outcome Measures (known as PROMs). The Office of Health Economics (Devlin and Appleby, 2010) argues that, if healthcare interventions aim to improve how participants feel about their health, it is the participants themselves who can provide the best source of information as to what improvements or changes might be attributable to the treatment. As a result, when children are properly asked, they can be the best source of information for their own assessments of health and wellbeing (Devlin and Appleby, 2010). Most recently, participant-reported instruments have been developed to measure health in terms of the effect of any given state of health on the ability to function and enjoy life, what is often referred to as measures of health-related quality of life (Foster et al., 2018). This was one of the main outcome measures used in this study.

Another challenge for school mental health services is that the focus relies heavily on the treatment of severe emotional problems or disorders, whereas early detection and prevention might be equally important for the promotion of children’s wellbeing (World Health Organisation [WHO], 2004; Goldie et al., 2016). When opportunities for prevention are missed, there are increased chances for children to drop out of school, self-harm, become aggressive, violent, or even suicidal. In the United Kingdom, it is estimated that 7,000 children are being excluded annually (equivalent to 35 children per day), while 1,300 of these exclusions come from primary schools only (Children’s Commissioner for England, 2017). In addition, approximately one in ten children experience at least one diagnosed mental health difficulty that may impact their education (McDonald and Holttum, 2020). Although a quarter of a million children in primary schools have support from mental health services (McDonald and Drey, 2018), the Children’s Society (2018) has estimated that 70% of children have not had appropriate interventions and supportive services at a sufficiently early age. Particularly, 28% of referrals are turned away immediately, while the waiting lists can take up to a year (Children’s Commissioner for England, 2017). Despite the growth of arts therapies in schools, delays in addressing children’s needs can have long lasting and potentially irreversible negative effects. It is therefore important that psychological support is offered at the early stages of children’s education.

The overarching aims of this pilot were: (a) to explore whether all components of the study (i.e., recruitment, randomization, and follow-up) can work together and run smoothly in a larger trial; and (b) to investigate the effectiveness of arts therapies on several quantitative, qualitative and arts-based outcome measures. This paper will specifically focus and present on the quantitative effects.

## Materials and Methods

The design of the current study was grounded on the findings of a systematic review (Moula et al., 2020) that informed the development of the sessions, the choice of outcome measures, and the research methods that were used. For example, the systematic review revealed the need for evidence of long-lasting effects of arts therapies, as well as the integration of quantitative, qualitative and arts-based research methods in experimental studies. Because of the complexity of health difficulties, complex social interventions often require data collection from various perspectives and methods (Johnson et al., 2007). Hence, a mixed methods methodology was needed (Creswell, 2014) to understand the outcomes of arts therapies both from children’s experiences and standardized measures.

The systematic review led to the development of a pilot designed to (a) explore whether all components of the study (i.e., recruitment, randomization, and follow-up) can work together and run smoothly in a larger trial; and (b) investigate the effectiveness of arts therapies on several quantitative, qualitative and arts-based outcome measures. This paper is focused on the quantitative effects of this model of arts therapies on a battery of standardized outcome measures relating to: health-related quality of life; wellbeing; life-functioning; emotional and behavioral difficulties; and sleep duration. All assessments were completed primarily by children, but also their teachers. The sustained impact of the intervention on these measures was evaluated at 3, 6, and 12-months follow-up. As the current study is a pilot, sample size calculations were performed to inform the design of future large scale randomized controlled studies. The primary outcome measures were Health Related Quality Of Life (HRQOL), assessed using the EQ-5D-Y (Wille et al., 2010), and the Child Outcome Rating Scale (CORS) (Low et al., 2012) which is a measure of wellbeing and psychosocial functioning. Both measures focus on the viewpoint of the individual and responses were given using a visual analog scale.

A cross-over randomized controlled design was employed in this study (Creswell, 2014). All participants were randomly allocated to the intervention or control groups in order to minimize group differences due to biased selection or other confounding variables (World Health Organisation [WHO], 2004). Half of the participants were assigned to the intervention immediately after the randomization, whereas the other half acted as the control group in the beginning (waiting-list) and received the intervention 3 months later. This approach had the advantage of reducing the sample required, since each participant served as both a participant in the intervention as well as a control. It also decreased the biological variability, which is inherent in comparing different participants, by comparing each participant with themselves (World Health Organisation [WHO], 2004).

The National Institute for Health Research (National Institute for Health Research [NIHR], 2017) defines pilot studies as “*a version of the main study that is run in miniature to test whether the components of the main study can all work together. It is focused on ensuring that the processes of the main study* (*e.g., recruitment, randomization, treatment, and follow-up assessments*) *all run smoothly*” (p. 2). However, certain elements can also be tested for feasibility (Craig et al., 2008). In this study these elements were: (i) the acceptability of the randomization process to schools; (ii) the implementation of the arts therapies protocol; and (iii) the methods used for process and outcome evaluation.

Prior to this study, two systematic reviews were carried out to synthesize the evidence from arts therapies interventions delivered in primary mainstream schools (Moula, 2020; Moula et al., 2020). The findings showed that for a pilot study, a minimum of 60 participants (30 in each group for a two-arm intervention) may be statistically meaningful (Uttley et al., 2015), which is also confirmed in relevant methodological texts (Browne, 1995; Murphy et al., 2014). In a recent systematic review examining the clinical effectiveness of art therapy in those with non-psychotic mental health disorders (Uttley et al., 2015) the mean sample size was also found to be 52 participants. In addition, for an anticipated large effect size (whereby Cohen’s *d* = 0.8), a power level of 0.8 and probability level of 0.5, a minimum sample size of 26 individuals per group is required (therefore 52 participants for a two-arm intervention as in the current study). Based on the above, this pilot study aimed to begin with 62 participants, giving each group a sample size of 31 individuals, as some attrition was expected to result in a reduced final sample size. Assuming the final sample consisted of at least 26 participants per group, this could allow for a definitive randomized controlled trial to be designed to yield a true power of 80%. Moreover, a cross-over design was applied with the control group becoming part of the treatment group; thereby increasing the sample size.

The current pilot served to acquire estimates of the variances of the primary dependent variables (DV’s) for power calculations. This helped to determine which measures should be used in a larger study. The minimal clinically important differences (MCIDs) were determined through a distribution-based approach, the effect size. The change in scores corresponding to the small effect size considered the MCID (Samsa et al., 1999; Taylor et al., 1999; Hays and Woolley, 2000; Kulkarni, 2006).

A design feature of this study is that those who took part in the control groups initially were “crossed-over” into the arts therapies intervention groups. However, those who took part in the arts therapies intervention groups were not “crossed-over” into the control groups due to the involvement of several follow-up measures. As a result, this study did not follow the typical cross-over design. A key reason for using the same participants in the intervention and control groups was an ethical decision to ensure that all children received equal opportunities and benefits from their participation in this study. There was no strong rationale for completing the cross-over design (i.e., crossing over the arts therapies intervention to receive the control condition) on the grounds that the effects of the treatment were anticipated to be long-term and hence the follow-up measures. The typical cross-over studies are also considered more appropriate where the effects of the treatment(s) are short-lived and reversible, which was not the case in the current study (Cleophas, 1990; Elbourne et al., 2002; Mills et al., 2009).

### Participants

Participants were 62 children aged 7–10 years. A detailed description of the recruitment and randomization methods, as well as the ethical considerations are available in the study protocol (Moula et al., 2019) and therefore only a brief summary is presented here. Participants were recruited from schools across the Northwest of England. Headteachers identified two classes in greater need for psychological support as children with mild emotional and behavioral difficulties were the targeted participant cohort. Children were randomly allocated to the intervention or control groups via a random number generator software^1^. Where 7/8 participants were of the same gender, the last participant was replaced by a participant of the different gender to avoid outgroup gender isolation. A total of four schools signed up to the study and each school administered a different form of arts therapies: music therapy, art therapy, dance movement therapy, or dramatherapy, to an equal number of participants. The list of eligibility criteria is presented in Table 1.

**TABLE 1:** Eligibility criteria for selecting participants in the study.

**Inclusion criteria:** • Children aged 5–12 years’ old. • Children who experience mild emotional and behavioral difficulties based. • On teachers’ ratings in the “Strengths and Difficulties Questionnaire with impact supplement for the teachers of 4–17 year olds.” • Children with a diagnosis of disabilities that are not expected to affect them in participating in group arts therapies, including, but not limited to, • Dyslexia, dysgraphia, dyscalculia, intellectual disabilities. • Children that both themselves and their parents or legal guardians consent to their participation in the study. • Children with or without fluency in English.
**Exclusion criteria:** • Children with a diagnosis of disabilities that are expected to need one-to-one support, including, but not limited to, autistic spectrum disorder, attention deficit hyperactivity disorder, cerebral palsy, Down syndrome, epilepsy, cystic fibrosis, spina bifida, multiple sclerosis, depression, anxiety disorder, hearing impairment, visual impairment. This exclusion was made on the basis that the arts therapists might not be able to meet children’s needs within the large groups of 6–8 children. • Children who would require one-to-one support for any other reason. • Children who are already involved in arts therapies.

### Arts Therapies Intervention

The systematic review (Moula et al., 2020) informed the development of the therapeutic protocol based on the evidence of ‘what works best’ for children at schools. This protocol defined the key therapeutic ingredients (Table 2) that were integrated into a pluralistic theoretical and therapeutic framework.

**TABLE 2:** Key therapeutic ingredients.

• Approaching children with warmth (Choi et al., 2008). • Developing an empathetic attitude (Rousseau et al., 2005). • Focus on establishing trusting relationships (Hilliard, 2001). • Working with children where they are (Deboys et al., 2017). • Revisiting past experiences with direct impact on the here and now (Hilliard, 2001). • Working with the group as a whole (Koshland et al., 2004). • Encouraging self-awareness (Koshland et al., 2004). • Facilitating the development of agency and the capacity to grow (Hilliard, 2001; Rousseau et al., 2005). • Developing useful coping mechanisms (Hilliard, 2001; Koshland et al., 2004; Choi et al., 2008). • Finding the balance between verbal and non-verbal communication (Rousseau et al., 2005).

Arts therapies were delivered once a week for 1 h, for eight consecutive weeks. The length of the intervention was tailored to fit within the available weeks of a school term. If the number of sessions exceeded 8 weeks, the sessions would have been interrupted by a long 2-week break (i.e., Christmas and Easter break), and time and resources would have been spent to allow the group to reconnect. In addition, the format of delivering eight sessions was found to be common practice according to the systematic review (Moula et al., 2020).

**FIGURE 1:**
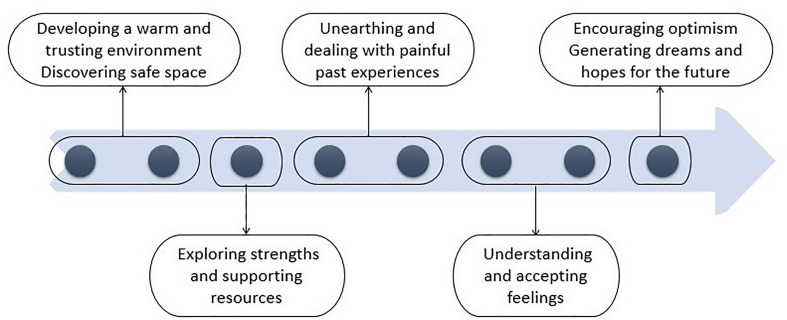
Therapeutic model.

Based on the therapeutic model (Figure 1), each session was focused on a specific topic and therapeutic goals, based on existing evidence of previous arts therapies interventions (Hilliard, 2001; Koshland et al., 2004; Karkou and Sanderson, 2006; Choi et al., 2008; Karkou, 2010). There were also strong influences from the project ‘Arts for the Blues,’ a study that supported the development of an evidence-based creative intervention for adults (Haslam et al., 2019; Parsons et al., 2019). Each session has been described thoroughly in the study protocol (Moula et al., 2019).

Arts therapists were trained on the protocol application prior to the study and were encouraged to make their own clinical judgments moderating the structure of the sessions when needed. These modifications were recorded for fidelity purposes. The protocol was used to ensure arts therapists adhered to the overall therapeutic model adopted in this study and to allow future replications. This was particularly important considering that all four disciplines of arts therapies were included.

### Outcome Measures

#### Questionnaires

The following three standardized questionnaires were administered: the Quality of Life scale for children (EQ-5D-Y) (Wille et al., 2010) and the Child Outcome Rating Scale (CORS) (Low et al., 2012), both of which were completed by the children. The Strengths and Difficulties Questionnaire (SDQ) (Goodman, 2001) was completed by the class teachers.

The Quality of Life scale for Children (EQ-5D-Y) (Wille et al., 2010) is a standardized quantitative measure of health-related quality of life (HRQOL) that reflects young people’s own judgment and can be used in a wide range of health conditions and treatments. The questionnaire is intended to evaluate the dimensions of activity level, looking after one’s self, doing usual activities (for example, going to school, hobbies, and sports), having pain or discomfort and feeling worried, sad or unhappy. An overall score is calculated for the five items included in the test, with scores ranging from 0 to 50. Test–retest reliability ranges between 69.8 and 99.7% (Ravens-Sieberer et al., 2010). Kappa coefficients reach up to 0.67, while the correlation coefficients with other measures of self-rated health have indicated convergent validity up to *r* = −0.56 (Ravens-Sieberer et al., 2010).

The Child Outcome Rating Scale (CORS) (Low et al., 2012) is a standardized measure used to evaluate areas of wellbeing and life functioning that might change as an outcome of an intervention. These areas relate to: personal or symptom distress (measuring individual well-being); interpersonal well-being (measuring how well the child is getting along in intimate relationships); social role (measuring satisfaction with work/school and relationships outside of the home) and overall well-being. Scores on this scale range from 0 to 40 with higher scores indicating an increase in functioning. Research on the validity and reliability of the test suggests: moderate to high reliability (Cronbach’s alpha = 0.93; Bringhurst et al., 2006), moderate test–retest reliability (*r* = 0.8; Bringhurst et al., 2006), and moderately strong concurrent validity (correlation coefficients range from 0.56 to 0.69; Bringhurst et al., 2006) with longer, more established measures of treatment outcome and therapeutic alliance (Bringhurst et al., 2006).

The Strengths and Difficulties Questionnaire (SDQ) (Goodman, 2001) with impact supplement for the teachers of 4–17 year’ olds is an emotional and behavioral screening questionnaire. The impact supplement examines the nature of a young person’s difficulties, such as burden to others, social impairment, chronicity, and distress related to a reported problem. High scores on this questionnaire reflect a greater degree of emotional and behavioral difficulties. Existing evidence suggests that this tool exhibits strong internal consistency (Cronbach’s alpha = 0.81; Yao et al., 2009); moderate test–retest reliability with correlation coefficients reportedly ranging from 0.51 to 0.75 (Lundh et al., 2008) and 0.71 (Yao et al., 2009), and good concurrent validity (correlation coefficients range from 0.49 to 0.73; Muris et al., 2003).

#### Sleep Duration

Sleep activity was assessed using activity trackers (Fitbits). The duration of sleep was calculated as the total number of minutes asleep minus the number of minutes awake during the night. FitBits were given to children for 3 days before the beginning of the sessions and for 3 days after the end of the sessions. Duration of sleep was averaged across the 3 days pre-intervention and the 3 days post-intervention.

### Procedure

**FIGURE 2:**
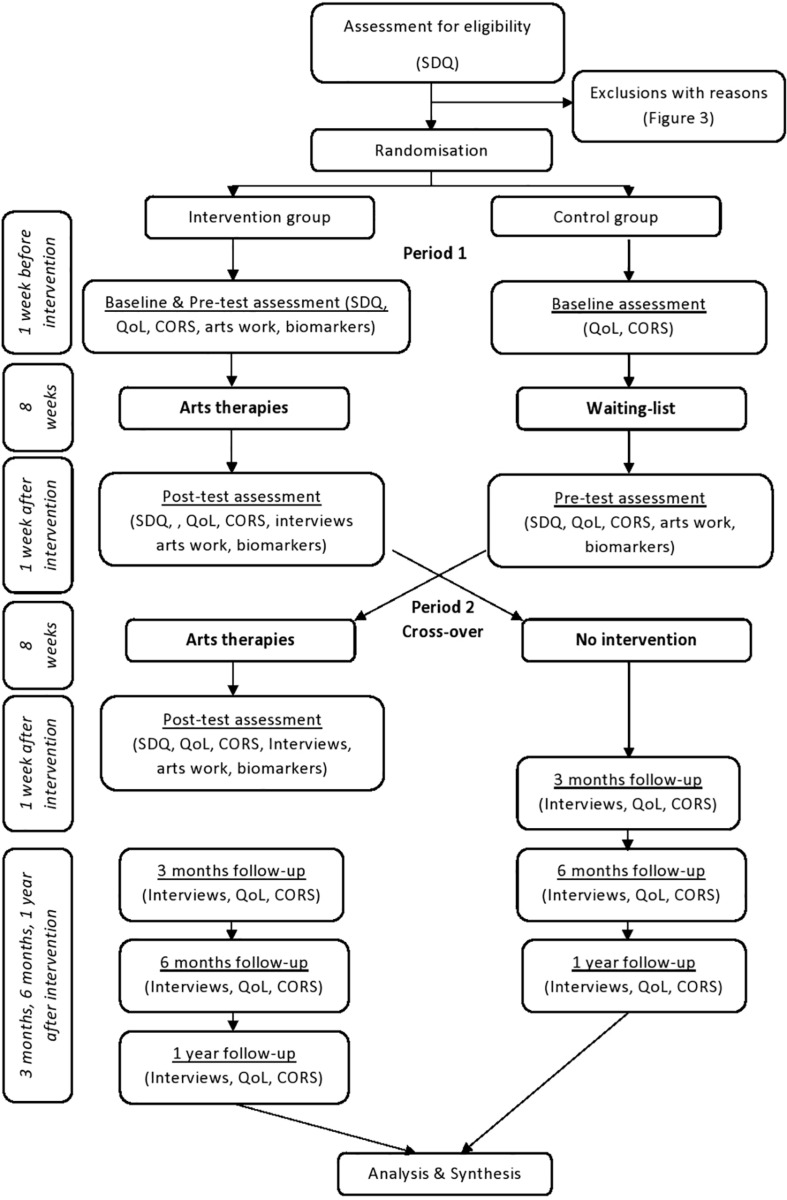
Procedure.

An overview of the procedure used in the study is presented in Figure 2, while further details are included in the study protocol (Moula et al., 2019). Briefly, following recruitment and randomization, a total of 31 participants were allocated to the initial intervention groups, and 31 participants were allocated to the control groups (waiting list). Following the intervention period, those on the waiting list were “crossed-over” from the control to the intervention groups. Therefore, children from the waiting list also received the intervention. Following dropouts (see Table 3) the cross-over design included a control group of 30 participants, and an intervention group of 56 participants.

**TABLE 3:** Recruitment and dropout rates.

**Type of arts therapies**	**No of children recruited**	**No of children completed**	**Dropouts occurred (group)**	**Dropout stage**	**Dropout justification**
AT	16	14	2 (from control)	During sessions	1 hospitalization 1 moved abroad
DT	16	14	2 (1 from each group)	Before sessions	2 did not wish to miss PE classes
DMT	16	16	–	–	–
MT	14*	12	2 (from intervention)	Before and during	1 left school 1 did not want to miss the assembly
**Total**	**62**	**56**	**6**		

*Seven (instead of eight) children per group were recruited in the music therapy group due to the music therapist’s concerns about the group size. AT, art therapy; DT, dramatherapy; DMT, dance movement therapy; MT, music therapy.

HRQOL and CORS were administered within 1 week prior and 1 week commencing the intervention period, referred to as the pre- and post-intervention measures, respectively. The SDQ and FitBit data were collected only before and after the arts therapies intervention (not the control period). A total of 11 children chose not to wear their Fitbits. HRQOL and CORS were also administered at 3-months, 6-months, and 1-year follow-up. The drop-out rate for follow-up data up to 6 months was 9% (*n* = 5). The Covid-19 outbreak occurred during the 1-year follow-up and as a result, data from 15 children were missed, specifically from the music and dance movement therapy groups.

### Statistical Analysis

All four arts therapies were considered together and composed the intervention group. Complete case analysis was performed for all outcome measures. The pattern of missingness within the data was assumed to be missing completely at random (MCAR), with missing data observed at follow-up spread across all arts therapies groups. ANCOVA’s were used to test for differences in HRQOL and CORS post-intervention scores between control and intervention groups before the cross-over (*N* = 58), while also controlling for pre-intervention scores which were entered into the models as covariates.

Repeated-measures ANOVAs were used to test for changes in pre-and post-intervention measures for HRQOL, CORS, duration of sleep and SDQ following cross-over (*N* = 56). A between groups factor was also entered in the models for HRQOL and CORS to determine whether the change pre-and post-intervention differed significantly between the intervention and control groups. Separate repeated-measures ANOVA’s were performed on the follow-up data to test for changes in HRQOL and CORS score between the post-intervention assessment, and the follow-up. Further repeated-measures ANOVA’s were performed to test whether the change in HRQOL and CORS score differed between the four arts therapies groups using data only from the intervention groups following cross-over. This was performed as exploratory analysis to determine whether the type of arts therapies affected the key outcome measures. Statistical analyses of ANCOVA’s and repeated measures ANOVA’s were performed using IBM’s SPSS (version 25). Statistical significance was determined using an alpha level of 0.05. Eta squared was calculated to determine effect size for all tests performed and then converted to Cohen’s *d* using free online software by Psychometrica (Lenhard and Lenhard, 2016). To calculate the change score equivalent to the MCID, the standard deviation of the baseline scores was multiplied by 0.2 (the small effect size) (Samsa et al., 1999). MCID was calculated for HRQOL and CORS before and after cross-over as well as for the duration of sleep and SDQ.

Sample size calculations were computed for HRQOL and CORS as the necessary sample size for a clinical trial using XLSTAT-Power^2^. Specifically, calculations were performed for a superiority trial, which aims to test whether one treatment is better than another. XLSTAT-Power can determine the sample size necessary to reach a given power. Sample size calculations were determined for a power of 0.8 and 0.9 using an alpha level of 0.05.

With regards to the missing data, there are multiple approaches which have been developed including, complete case analysis, mean substitution, last observation carried forward, intention to treat (ITT) analysis, and variants of the multiple imputation approach. The intention to treat (ITT) principle is widely employed and reports of ITT or modified ITT were found to address outcomes in 85% of the studies reviewed by Bell et al. (2014). ITT protects against bias by preserving the benefits of randomization (Hollis and Campbell, 1999; Gravel et al., 2007). However, when outcome data is missing, a true ITT analysis can be difficult or impossible to achieve and researchers must make assumptions which might be unreliable, while the ability to draw conclusions about a causal link is also compromised (Altman, 2009; Can et al., 2011; White et al., 2011; Dziura et al., 2013; Bell et al., 2014). The current study employed a complete case analysis approach which involves including only subjects with a complete set of outcome data. The approach is computationally simplistic and, moreover, the estimates of treatment differences are unbiased when missing data are completely at random (MCAR), as is the case in the current study (Dziura et al., 2013). Whilst the payoff is a reduction in sample size, the alternative method to address missing data that involve estimating outcomes in the absence of actual data suffer the problem of introducing uncertainty about what really happened to the participants (Streiner and Geddes, 2001). Given that the current study was a pilot, with the primary goal being to accurately estimate treatment differences in order to calculate sample sizes for the outcome measures for a full randomized controlled study, a complete case analysis approach was considered more appropriate.

## Results

**FIGURE 3:**
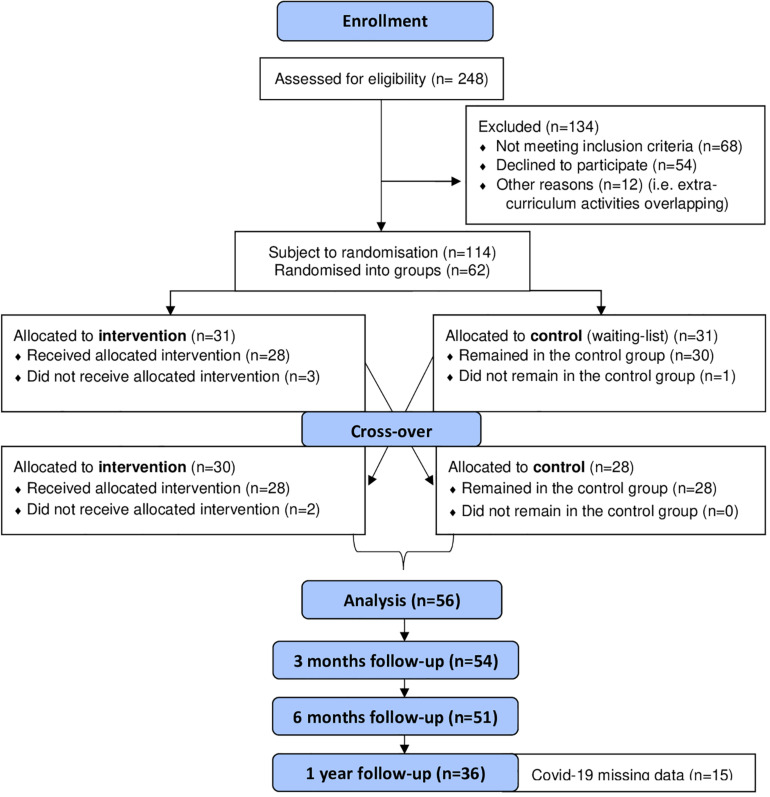
CONSORT 2010 flow diagram.

The CONSORT flow diagram (Figure 3) illustrates the enrolment of participants, their allocation to treatment, follow-up, and data analysis. Descriptive statistics, including means and standard deviations for HRQOL and CORS are shown in Table 4. The mean scores revealed that the pre- and post-intervention HRQOL scores for the intervention groups *before* cross-over were almost identical, but with a larger variation in scores post-intervention; whereas the mean for CORS was greater post-intervention than pre-intervention for the intervention groups before cross-over. Descriptive statistics (Table 4) showed greater post-intervention means for the intervention groups compared to the control groups for both HRQOL and CORS *following* cross-over.

**TABLE 4:** Descriptive statistics including means and standard deviations for HRQOL and CORS scores pre- and post-intervention, for the total sample and separated by control and intervention groups before and following cross-over design.

	**HRQOL pre-intervention**	**HRQOL post-intervention**	**CORS pre-intervention**	**CORS post-intervention**
Control (*N* = 30)	31.29 (9.63)	31.91 (10.5)	29.67 (7.95)	30.67 (7.59)
Intervention *before* cross-over (*N* = 28)	36.60 (7.92)	36.48 (10.33)	31.24 (7.25)	34.14 (6.84)
Total *before* cross-over (*N* = 58)	33.90 (9.16)	34.12 (10.58)	30.44 (7.59)	32.34 (7.38)
Total *after* cross-over (*N* = 56)	34.25 (9.68)	35.98 (10.19)	30.55 (7.35)	34.41 (6.69)

### Health Related Quality of Life

#### Before Cross-Over

**FIGURE 4:**
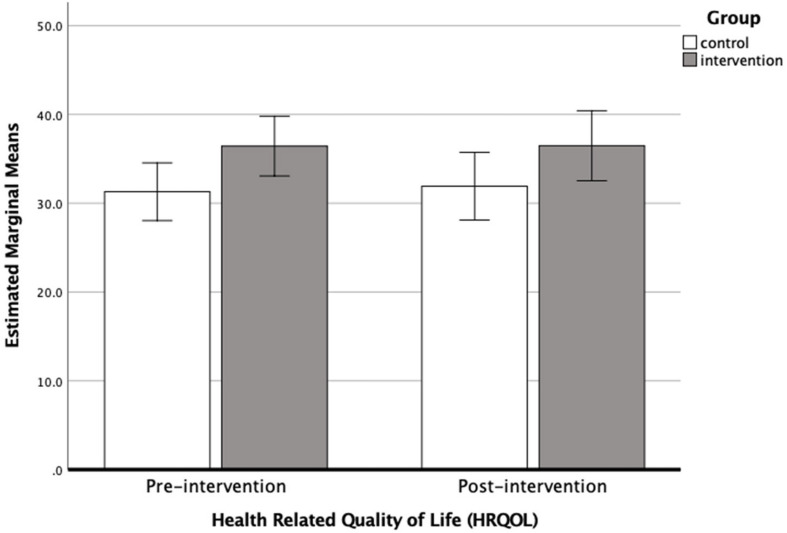
Bar chart showing the estimated marginal means (with 95% confidence intervals) for HRQOL scores pre-intervention and post-intervention before cross-over (i.e., when *N* = 58), for the control (*N* = 30) and intervention groups (*N* = 28).

Results from the ANCOVA showed that pre-intervention HRQOL score was a significant predictor of post-intervention score across all participants (*F*_1_,_55_ = 40.620, *P* < 0.001, η^2^ = 0.035, Cohen’s *d* = 0.38). However, no significant effect was found between the groups (*F*_1_,_55_ = 0.085, *P* > 0.05, η^2^ = 0.0001, Cohen’s *d* = 0.02). A bar chart showing the estimated marginal mean scores with 95% confidence interval error bars for HRQOL scores pre- and post-intervention is presented in Figure 4.

#### Following Cross-Over

A repeated-measures ANOVA was performed on the HRQOL data *following* cross-over to test for a significant change between pre- and post- intervention period and to determine whether this change differed between the control (*N* = 30) and intervention (*N* = 56) groups. Results showed the change in HRQOL following the intervention period was not significant (Wilks’ Lambda = 0.986, *F*_1_,_84_ = 1.036, *P* > 0.05, η^2^ = 0.012, Cohen’s *d* = 0.22), nor did the change differ significantly between intervention and control groups (Wilks’ Lambda = 0.997, *F*_1_,_84_ = 0.232, *P* > 0.05, η^2^ = 0.003, Cohen’s *d* = 0.11). Parameter estimates revealed no significant difference in HRQOL score between intervention and control groups pre-intervention (coefficient = −2.953, *SE* = 2.187, *P* = 0.2, 95%CI: −7.302, 1.396) or post intervention (coefficient = −4.069, *SE* = 2.331, *P* = 0.09, 95%CI: −8.70, 0.57).

#### Minimal Clinically Important Differences (MCIDs)

The mean difference between pre- and post-intervention score was less than the MCID for the intervention groups before cross-over (i.e., 0.12 < 1.58) and following cross-over (i.e., 1.55 < 1.94), indicating that the minimal clinically important difference was not been obtained for a small effect size for HRQOL.

#### Sample Size

Sample size calculations, determined using XLSTAT-Power on HRQOL before cross-over, indicated that a sample size of 168 participants (84 participants per group) would be required to achieve 0.8 power and 225 participants (113 participants per group) to achieve 0.9 power.

#### Follow-Up Analysis

**FIGURE 5:**
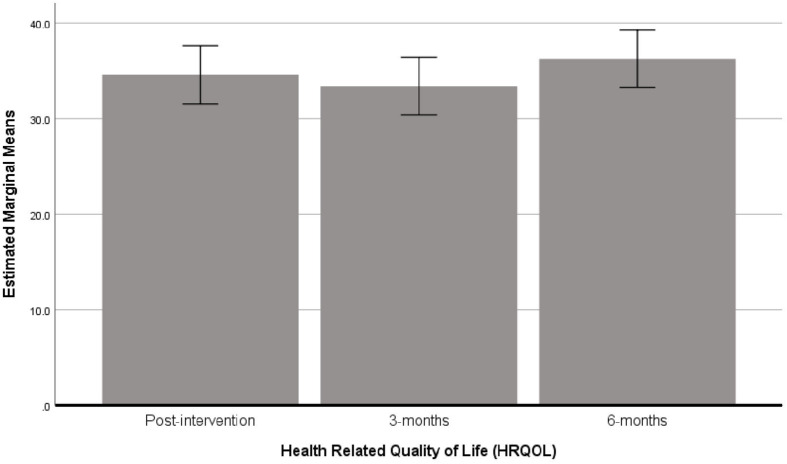
Bar chart showing the estimated marginal means (with 95% confidence intervals) for HRQOL scores post-intervention, 3 and 6 months *following* cross-over (i.e., when *N* = 56).

Mean scores indicated a small fluctuation in HRQOL score between follow-up points which drop from 34.59 (*SD* = 10.72) immediately post-intervention to 33.39 (10.62) at 3-months, but then increased past post-intervention assessment at 6-months to 36.24 (*SD* = 10.57) (Figure 5). Results from the repeated measures ANOVA showed no significant change in HRQOL score between post-intervention and follow-up measures (Wilks’ Lambda = 0.944, *F*_2_,_48_ = 1.414, *P* > 0.05, η^2^ = 0.028, Cohen’s *d* = 0.34).

The follow-up data at 12-months post-intervention was collected from all but two of the arts therapies groups (six out of the eight groups). The missing data were from the children who took part in the second cohort of music therapy and dance movement therapy. This data was missing due to the unforeseen circumstances resulting from the pandemic COVID-19. The 12-month follow up outcome data for HRQOL was collected from a sample of 36 participants. Two further repeated measures ANOVA’s were performed to test the change in HRQOL scores between 6- and 12-months in these participants. Descriptive statistics showed that the mean scores for HRQOL between 6- and 12-months were very similar (i.e., 35.35 ± 10.3 and 35.44 ± 9.4, respectively), and that this change was not significant (*F*_1,35_ = 0.004, *P* > 0.05, η^2^ = 0.0001, Cohen’s *d* = 0.02).

#### Arts Therapies

**FIGURE 6:**
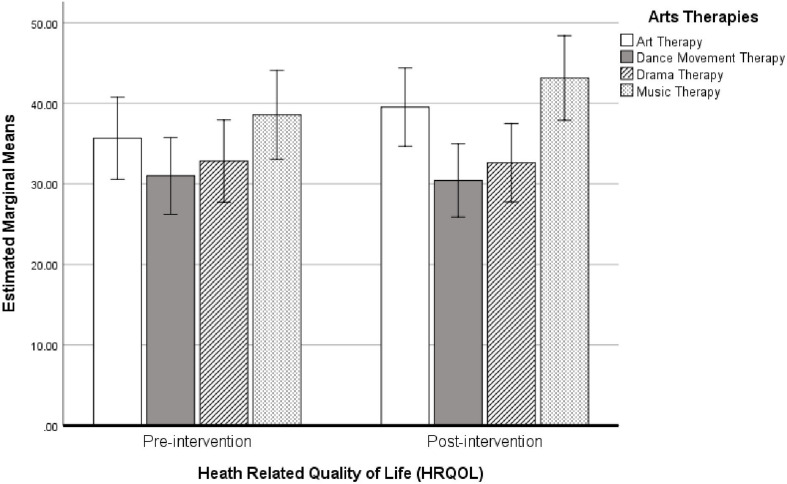
Bar chart showing the estimated marginal means (with 95% confidence intervals) for HRQOL scores pre- and post-intervention *following* cross-over, separated by arts therapies groups (*N* = 56).

Means and standard deviations for HRQOL score pre-and post-intervention for each of the arts therapies groups is shown in Table 5. Results from the repeated-measures ANOVA showed no significant change in HRQOL pre- and post-intervention overall (Wilks’ Lambda = 0.972, *P* > 0.05, *F*_1_,_52_ = 1.486, η^2^ = 0.027, Cohen’s *d* = 0.33). Moreover, the change in HRQOL score did not differ between the arts therapies groups (Wilks’ Lambda = 0.960, *P* > 0.05, *F*_3_,_52_ = 0.723, η^2^ = 0.039, Cohen’s *d* = 0.4). A bar chart showing the estimated marginal mean scores with 95% confidence interval error bars for HRQOL scores pre- and post-intervention, separated by arts therapies groups, are shown in Figure 6. Inspection of the means revealed that the mean difference between pre- and post-intervention measures was larger for those in music therapy (4.59) and art therapy (3.86) than those doing either dance movement therapy (−0.56) or drama therapy (−0.21).

**TABLE 5:** Descriptive statistics including means and standard deviations for HRQOL and CORS scores pre- and post-intervention, separated by arts therapies group *following* cross-over design (*N* = 56).

	**HRQOL pre-intervention**	**HRQOL post-intervention**	**CORS pre-intervention**	**CORS post-intervention**
Art therapy (*N* = 14)	35.67 (10.19)	39.53 (9.26)	32.86 (6.57)	36.71 (5.40)
Dance movement therapy (*N* = 16)	30.99 (10.06)	30.43 (8.82)	25.88 (8.14)	32.63 (6.53)
Dramatherapy (*N* = 14)	32.84 (8.92)	32.63 (8.32)	30.36 (5.90)	32.43 (7.29)
Music therapy (*N* = 12)	38.57 (8.55)	43.16 (9.95)	34.33 (5.76)	36.42 (6.89)

### Child Outcome Rating Scale

#### Before Cross-Over

**FIGURE 7:**
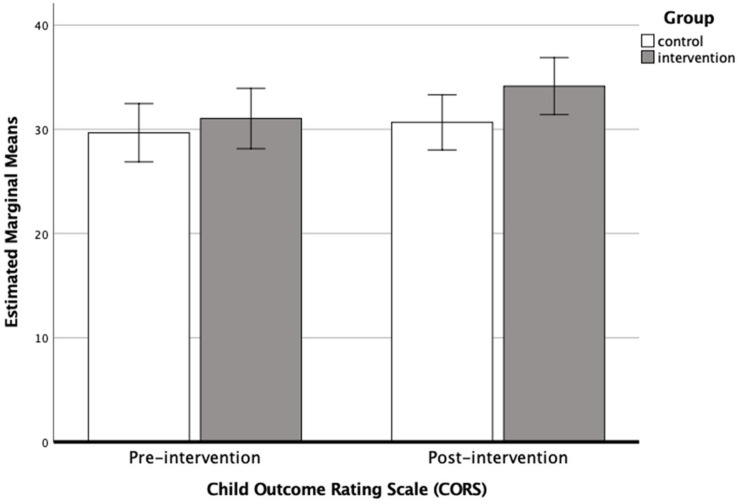
Bar chart showing the estimated marginal means (with 95% confidence intervals) for CORS scores pre-intervention and post-intervention *before* cross-over, (i.e., when *N* = 56) for the control (*N* = 30) and intervention groups (*N* = 28).

An ANCOVA was used to determine differences in post-intervention CORS score between intervention and control groups while controlling for pre-intervention CORS score using data *before* cross-over. Results showed that pre-intervention CORS score was a significant predictor of post-intervention CORS score (*F*_1_,_55_ = 43.116, *P* < 0.001, η^2^ = 0.02, Cohen’s *d* = 0.29). No significant difference was found for group (*F*_1_,_55_ = 3.291, *P* = 0.08, η^2^ = 0.002, Cohen’s *d* = 0.08). A bar chart showing the estimated marginal mean scores with 95% confidence interval error bars for CORS scores pre- and post-intervention are shown in Figure 7.

#### Following Cross-Over

Repeated measures ANOVA were used to test for the change in CORS score following intervention. Results showed the change in CORS score was significant across all participants (Wilks’ Lambda = 0.902, *F*_1_,_84_ = 9.098, *P* = 0.003, η^2^ = 0.094, Cohen’s *d* = 0.64). The change in CORS score did not differ significantly between intervention and control groups (Wilks’ Lambda = 0.964, *F*_1_,_84_ = 3.148, *P* = 0.08, η^2^ = 0.033, Cohen’s *d* = 0.37). Parameter estimates revealed no significant difference in pre-intervention CORS score between intervention and control groups (coefficient = −0.887, *SE* = 1.71, *P* = 0.6, 95%CI: −4.29, 2.52). However, a significant difference in post-intervention CORS score was found (coefficient = −3.744, *SE* = 1.59, *P* = 0.02, 95%CI: −6.90, −0.59), with the intervention groups showing a significantly higher CORS score than the control groups. This suggests that while pre-intervention scores showed no significant difference between groups, following intervention CORS scores diverged with the intervention groups showing the greater improvement.

#### Minimal Clinically Important Differences (MCIDs)

The mean difference between pre- and post-intervention score was greater than the MCID for the intervention groups before cross-over (i.e., 2.9 > 1.45) and following cross-over (i.e., 3.86 > 1.47), indicating that the MCID has been obtained for a small effect size for CORS score within the intervention groups.

#### Sample Size

Sample size calculations, determined using XLSTAT-Power on CORS before cross-over scores, indicated a sample size of 142 participants (71 participants per group) would be required to achieve 0.8 power and 190 participants (80 participants per group) to achieve 0.9 power.

#### Follow-Up Analysis

**FIGURE 8:**
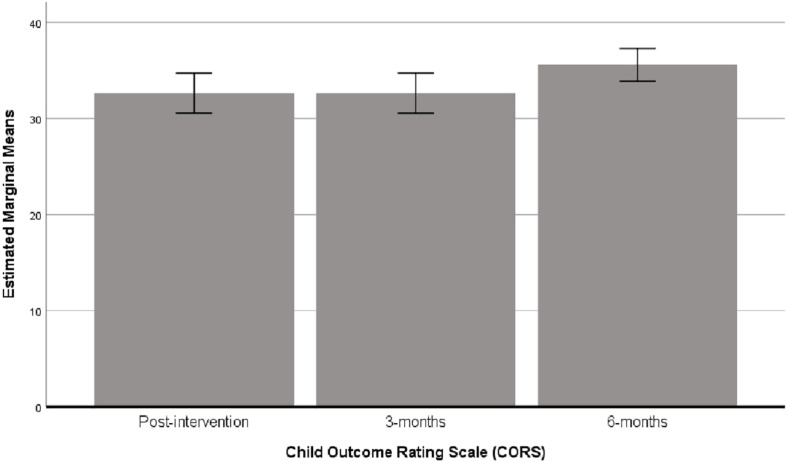
Bar chart showing the estimated marginal means (with 95% confidence intervals) for CORS scores at post-intervention, 3 and 6 months following cross-over (i.e., when *N* = 56).

Mean scores for CORS showed no change between post-intervention (mean = 32.63, *SD* = 7.37) and 3-months follow-up (mean = 32.63, *SD* = 7.37), though there was an increase at 6-months (mean = 35.58, *SD* = 6.07) (Figure 8). Results from the repeated measures ANOVA showed that this change in CORS score between assessment points was significant (Wilks’ Lambda = 0.842, *F*_2_,_49_ = 49.0, *P* = 0.015, η^2^ = 0.067, Cohen’s *d* = 0.54), with the significant change being between follow-up periods from 3- to 6-months.

As mentioned above, there were missing data at the 12-month follow-up due to COVID-19. The follow-up outcome data for CORS was collected from 36 participants. Two further repeated measures ANOVA’s were performed to test the change in CORS scores between 6- and 12-months in these participants. Descriptive statistics showed that the mean scores for CORS between 6- and 12-months were similar (i.e., 34.43 ± 6.7 and 33.14 ± 6.8, respectively), and that this change was not significant (*F*_1,35_ = 1.286, *P* > 0.05, η^2^ = 0.034, Cohen’s *d* = 0.38).

#### Arts Therapies

**FIGURE 9:**
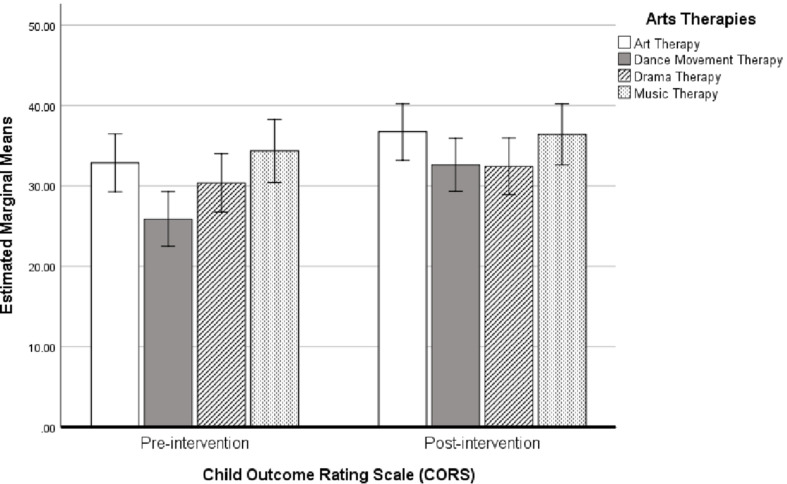
Bar chart showing the estimated marginal means (with 95% confidence intervals) for CORS scores pre- and post-intervention *following* cross-over, separated by arts therapies groups (*N* = 56).

Means and standard deviations for CORS score pre-and post-intervention for each of the arts therapies groups is shown in Table 5. Results from the repeated-measures ANOVA showed a significant change pre- and post-intervention overall (Wilks’ Lambda = 0.813, *P* = 0.01, *F*_1_,_52_ = 11.987, η^2^ = 0.178, Cohen’s *d* = 0.93). The change in CORS score did not differ between the arts therapies groups (Wilks’ Lambda = 0.938, *P* > 0.05, *F*_3_,_52_ = 1.145, η^2^ = 0.051, Cohen’s *d* = 0.46). A bar chart showing the estimated marginal mean scores with 95% confidence interval error bars for CORS scores pre- and post-intervention separated by arts therapies groups are shown in Figure 9. Mean differences between pre- and post-intervention revealed that all groups showed an improvement in CORS score, however, the change in score was larger for those engaged in dance movement therapy (6.75) and art therapy (3.85), than either dramatherapy (2.07) or music therapy (2.09).

### Strengths and Difficulties Questionnaire

A repeated-measures ANOVA showed a significant change in SDQ score following the intervention (*F*_1_,_58_ = 7.650, *P* = 0.008, η^2^ = 0.117). SDQ scores were significantly lower following the intervention (mean = 8.54, *SD* = 5.95) compared to before the intervention (mean = 9.76, *SD* = 6.06), indicating that teachers perceived a significant decrease in children’s emotional and behavioral difficulties. The change in mean score between pre- and post-intervention measures (i.e., 1.22) was larger than the MCID (i.e., 1.19).

### Duration of Sleep

A repeated measures ANOVA was performed to test for a change in duration of sleep pre- and post-intervention. Results showed a significant change in the number of minutes that participants slept (*F*_1_,_47_ = 10.372, *P* = 0.002, η^2^ = 0.181), with participants sleeping significantly longer post-intervention (mean = 489.79, *SD* = 46.02) compared to pre-intervention (mean = 464.40, *SD* = 71.60). Children were sleeping, on average, 25 min longer following the intervention. This marks a change from sleeping approximately 7 h and three-quarters pre-intervention to approximately 8 h and 10 min post-intervention. The MCID (i.e., 9.20) was less than the change in pre- and post-intervention scores (i.e., 25.39) indicating that the MCID had been reached.

## Discussion

One of the primary aims of this pilot study was to determine the sample size necessary to test the effectiveness of the arts therapies intervention on standardized outcome measures assessing quality of life, wellbeing and life functioning (assessed using the EQ-5D-Y and CORS), and to determine the lasting effect of the intervention on these measures at follow-up. The current study also aimed to assess the effect of the intervention on sleep duration (assessed using Fitbits) and emotional and behavioral difficulties (assessed by teachers using the SDQ).

This study found that the arts therapies did have a positive effect on the outcome measures. Specifically, an increase in scores on CORS was found following the arts therapies intervention. Although the change in CORS score did not differ significantly from the control groups, there was a trend toward significance (i.e., *P* = 0.08). Moreover, the minimal clinically important difference (MCID) was exceeded by double the required amount indicating that the arts therapies intervention was having a clinically significant effect on life functioning in the participating children. The same was not true for the outcome measure HRQOL where the MCID was not reached.

Previous research has suggested that arts therapies can be significantly better than the control groups for children’s quality of life (i.e., Beebe et al., 2010; Madden et al., 2010; Abdulah and Abdulla, 2018; Uggla et al., 2018). However, the evidence from the results of this study is inconclusive. Although there were improvements compared to the baseline, these were neither statistically nor clinically significant differences between the intervention and control groups. Some previous randomized controlled trials found similar results (Broome et al., 2001; Porter et al., 2017), although none of these studies used HRQOL as a primary outcome measure. Moderate effects for children’s quality of life were also found in systematic reviews (Bradt et al., 2015; Uttley et al., 2015), and meta-analyses (Koch et al., 2014, 2019). However, these reviews included children as well as young people and adults. Since there is no systematic review so far looking specifically at the effects of arts therapies for children’s quality of life, the existing evidence remains inconclusive.

Nonetheless, the present study indicated that following cross-over there were some improvements in HRQOL scores. Specifically, those engaged in the arts therapies interventions showed a greater improvement in HRQOL scores than those in the control groups. Although this difference was not significant, a small effect size as assessed by Cohen’s *d* (i.e., 0.22) was found. As Styles and Torgerson (2018) argue, when rigorous evaluations are performed using randomized experiments, the resulting effect sizes are often much smaller than previous research in the same substantive area, which might explain the small effect size here. This gives more weight to the current findings, particularly with regards to the results for CORS, where a small to moderate effect size (Cohen’s *d* = 0.37) was found for the difference between control and intervention groups *following* cross-over.

Although HRQOL has been used extensively in public health research because of its potential to capture the participants’ views on their own health (World Health Organisation [WHO], 2020), it has been criticized for lacking specificity given that the questions are designed to be applicable for participants with completely different conditions (Gibbons et al., 2016). Therefore, this measure might be successful in capturing views on general aspects of life (i.e., difficulties in moving or doing usual activities), but less successful capturing domains that are relevant to particular interventions (Foster et al., 2018); such as the effects on children’s emotional and behavioral difficulties, or even their overall sense of wellbeing and social functioning. This is why this measure was not used as a stand-alone tool in this study, but alongside the children’s outcome measurement scale, the teacher-reported questionnaire (i.e., SDQ) and the biomarkers.

The teacher-reported questionnaire of children’s strengths and difficulties (SDQ) showed a statistically significant change before and after the intervention, with children presenting fewer emotional and behavioral difficulties following the arts therapies intervention. These findings echo the results from previous studies (for example, Montello and Coons, 1999; Baker and Jones, 2005; Chong and Kim, 2010; Abdulazeem, 2014), which also concluded that teachers noticed significantly significant reduction in children’s emotional, conduct, hyperactivity and peer relationships problems as well as significant improvements in children’s prosocial behavior. These findings suggest the possibility that teachers can be perceptive of changes that children themselves might be unaware of, a result also found in relevant literature (Karkou, 2010). However, it needs to be noted that the teachers were not blind to the random assignment into the intervention or the control groups. The knowledge of who attended which group at what time might have partially affected the results. It is possible that teachers were expecting to see changes in the children who participated in the arts therapies groups.

The significantly positive changes in children’s quality of sleep was also important considering the impact of sleep health on both physical and psychological wellbeing. Particularly, sleep health is considered a major influential factor of neurologic function that helps to maintain wellness, increase resilience (Raven et al., 2018), and even change the perceptions of pain (Mondanaro et al., 2017). This is because neurologically, the mind remains active during sleep, unconsciously processing thoughts that have been put aside or dismissed (Feld and Born, 2017). Current evidence suggests that arts therapies can soothe and sedate (Loewy, 2020), increasing comfort and decreasing anxiety impeding sleep capacity (Lund et al., 2020). Although this is the first study to explore changes in sleep in children, there is preliminary evidence showing that arts therapies help improve sleep quality in adults (Wang et al., 2014; Kavurmaci et al., 2019) as well as in infants and toddlers (Loewy, 2020); while most of the evidence so far comes from studies in music therapy.

The systematic review that informed the development of this pilot (Moula et al., 2020) showed that only one out of the six experimental studies that were included explored the sustained outcomes of arts therapies (Abdulazeem, 2014). The lack of follow-up procedures or short follow-up periods may underestimate the potential benefits of treatment and fail to detect hazards, both of which can take longer to emerge (Llewellyn-Bennett et al., 2016). In addition, the value of the trial in understanding the efficacy and outcomes of an intervention may be limited (Llewellyn-Bennett et al., 2016). Long-term follow-up is important to ascertain the strength of the treatment over an extended period and the impact of change on participants’ life in the long term (Salkind, 2010). This study found that at follow up HRQOL scores were relatively stable following the intervention, with scores increasing slightly (albeit not significantly) at 6-months follow-up. CORS scores were stable at 3-months following the intervention, with a significant increase in scores at 6-months. This suggests that, on the basis of these two outcome measures, the intervention had a long-lasting impact. It is also possible that life functioning can improve 6 months after the intervention has ended, suggesting that positive changes can also become apparent after some time.

The effect of the type of arts therapies on the two key outcome measures showed that the change in HRQOL and CORS following the intervention did not differ significantly between the different arts therapies groups. This can be explained by the fact that, except for minor modifications, all arts therapies groups followed the same therapeutic approach and protocol. In statistical terms, these findings also suggest that all arts therapies interventions were equally effective regardless of the school environment, the arts therapists who delivered the sessions, or the arts media used.

The sample size calculations indicated that in order to achieve 0.8 power, HRQOL requires a minimum of 168 participants (i.e., 84 participants per group on the basis that there are two groups: one receiving the arts therapies intervention and the other the control condition), while CORS requires 142 participants (71 participants per group). To achieve 0.9 power, HRQOL requires a sample size of 225 participants (113 participants per group), and CORS a sample size of 190 participants (80 participants per group). Therefore, the sample size required for a trial is minimum 168 participants, or maximum 225 participants. Our systematic review (Moula et al., 2020) revealed that the largest sample size that has been employed so far in previous studies with these population characteristics and this kind of interventions was 138 participants (Rousseau et al., 2005), while the most common sample size ranged between 40 to 60 participants in total (Koshland et al., 2004; Choi et al., 2008; Abdulazeem, 2014) – although there were no indications as to whether sample size calculations had been performed prior to the recruitment of participants in these studies.

### Strengths and Limitations

This pilot study was developed based on the evidence that was collected and analyzed thoroughly in two systematic-reviews (Moula, 2020; Moula et al., 2020); which was an important strength of the study. The therapeutic protocol was also developed through a synthesis of previous interventions that had been successfully implemented and assessed by children themselves. It was also informed by other evidence-based arts therapies protocols (Haslam et al., 2019; Parsons et al., 2019). The detailed description of the therapeutic protocol (Moula et al., 2019), and the process evaluation from the perspectives of children, researchers and arts therapists in a forthcoming publication (Moula et al., in preparation), are expected to make this intervention replicable in future studies.

In addition, the data were collected through standardized outcome measures with a well-established validity and reliability. The power size calculation may inform future larger studies and the follow-up data may offer valuable information into the long-term outcomes of arts therapies. These aspects of the pilot are expected to contribute to the public recognition and inclusion of arts therapies in Cochrane reviews as well as in national and international guidelines. Setting children’s perspectives at the heart of the outcomes evaluation may also result in better-informed policies and practice decisions that are aligned to children’s needs and priorities.

Participant reported outcome measures (PROMs) are typically susceptible to missing data in randomized controlled studies as they depend on participants’ willingness and ableness to complete the measures (Rombach et al., 2016). This becomes especially problematic at follow-up, where it is often impossible to obtain complete data for all participants (Altman, 2009). In the current study, the rates of missing data were in the order of 2–3.5% for the primary outcome measures and 9% for follow-up analysis. This suggests that the overall rate of missing data was reasonably low.

The primary aim of this pilot study was to explore whether all components of the study can work together in a larger study (Moula et al., in preparation), while the investigation of the effectiveness on the measures of quality of life and wellbeing was the secondary aim. It is important to reiterate this as the relatively small sample size limits the conclusions drawn from the quantitative results and the generalizability of the findings. The limitations that are specifically linked to the intervention and process evaluation will be discussed in depth in the forthcoming publication (Moula et al., in preparation).

## Conclusion

The improvements in children’s HRQOL and CORS, although not statistically significant, were greater in those engaged in the arts therapies intervention than the control groups and were maintained at the follow-up stages, until 1 year post-intervention. Furthermore, results for the CORS did show a trend toward significance (*P* = 0.08) and actual significance following the cross-over (*P* = 0.003). Significant improvements were found for duration of sleep, assessed using activity trackers (i.e., Fitbits) and children’s emotional and behavioral difficulties, assessed by their teachers (SDQ). Minimal clinically important differences (MCID) were found for the outcome measures CORS, SDQ, duration of sleep, but not HRQOL. These results indicated that the arts therapies interventions were having a clinically significant effect on life functioning, duration of sleep, emotional and behavioral difficulties in the participating children. The findings also indicated that the intervention had a small positive effect on children’s quality of life. Overall, arts therapies may be more effective at addressing life functioning, which is inclusive of interpersonal relationships, as compared to quality of life. However, the study does suggest that all outcome measures assessed here would be suitable for inclusion in a larger study utilizing these arts therapies interventions. The power calculations suggested that, should these outcome measures be used in a study with a randomized controlled trial design, a sample size of 225 participants (or 113 per group) would be required to achieve a power of 0.9. Considering that this was a pilot study based on a relatively small sample size, additional research could be conducted to further explore these findings.

## Data Availability Statement

The raw data supporting the conclusions of this article will be made available by the authors, without undue reservation, to any qualified researcher.

## Ethics Statement

The studies involving human participants were reviewed and approved by Edge Hill University Ethics Panel. Written informed consent to participate in this study was provided by the participants’ legal guardian/next of kin.

## Author Contributions

ZM: conceptualization, methodology, investigation, analysis, writing – original draft, and visualization. JP: conceptualization, methodology, analysis, writing – review and editing, supervision, and visualization. VK: conceptualization, methodology, writing – review and editing, and supervision. All authors contributed to the article and approved the submitted version.

## Conflict of Interest

The authors declare that the research was conducted in the absence of any commercial or financial relationships that could be construed as a potential conflict of interest.

## Footnotes

1 https://www.random.org/

2 https://www.xlstat.com/en/
